# P-88. Trends in Osteomyelitis-Associated Mortality in the United States, 1999-2020: A Population-Based Analysis

**DOI:** 10.1093/ofid/ofaf695.317

**Published:** 2026-01-11

**Authors:** Krishna Sanaka, Andrew Wang, Marcos Schechter

**Affiliations:** University of Pennsylvania, Cleveland, OH; Geisel School of Medicine at Dartmouth, Lebanon, NH; Emory University School of Medicine, Atlanta, GA

## Abstract

**Background:**

Osteomyelitis is a serious infection associated with prolonged hospitalizations, amputation risk, and high mortality in vulnerable populations. Despite clinical advances, little is known about national trends in osteomyelitis mortality over time or across demographic groups. We aimed to characterize population-level mortality patterns associated with osteomyelitis in the United States from 1999 to 2020.

**Figure:**
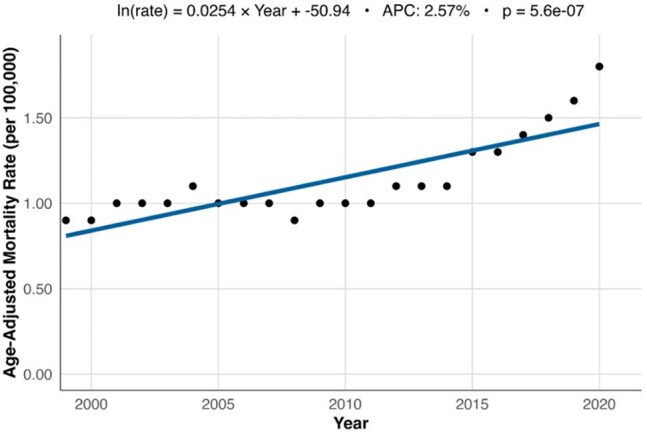
National Trend in Osteomyelitis Mortality, United States 1999-2020 Line graph showing the annual trend in overall age-adjusted mortality rates (AAMRs) for osteomyelitis from 1999 to 2020. AAMRs increased steadily over the study period, nearly doubling from the initial baseline.

**Figure:**
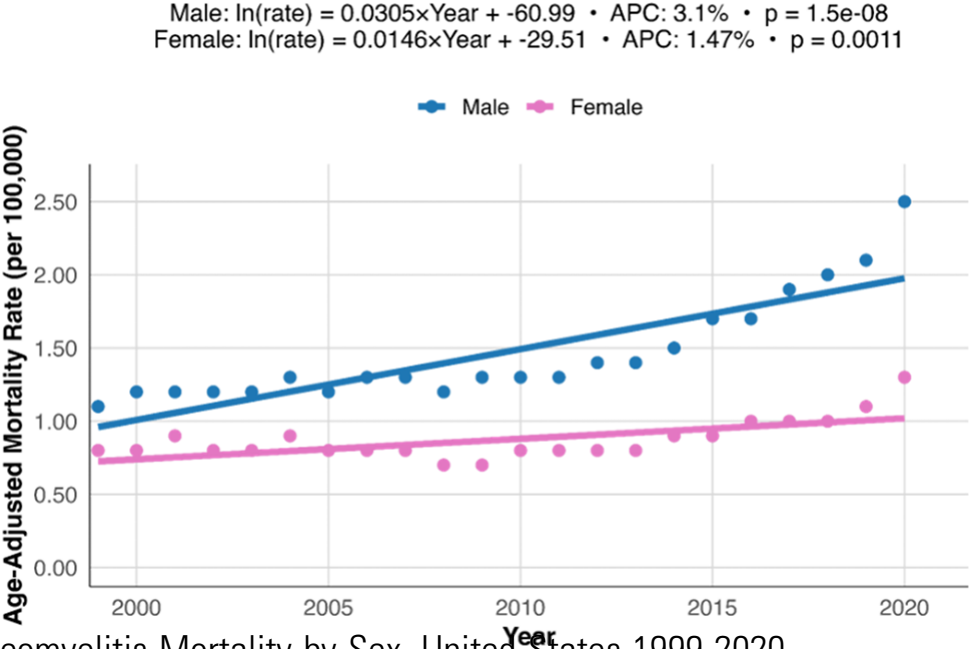
Trends in Osteomyelitis Mortality by Sex, United States 1999-2020 Scatter plot and fitted regression lines illustrating trends in AAMRs for osteomyelitis stratified by sex. Male individuals exhibited a steeper increase in mortality rates over time compared to female individuals.

**Methods:**

We conducted a retrospective, population-based analysis from the CDC WONDER Multiple Cause of Death database, covering all United States deaths from the years 1999 to 2020. Death certificates with osteomyelitis as an underlying or contributing cause were identified using the M86 code in the ICD-10. We fitted log-linear regression models to estimate the annual percent change (APC) in age-adjusted mortality rates (AAMRs) over time with stratifications for demographic subgroups.

**Figure:**
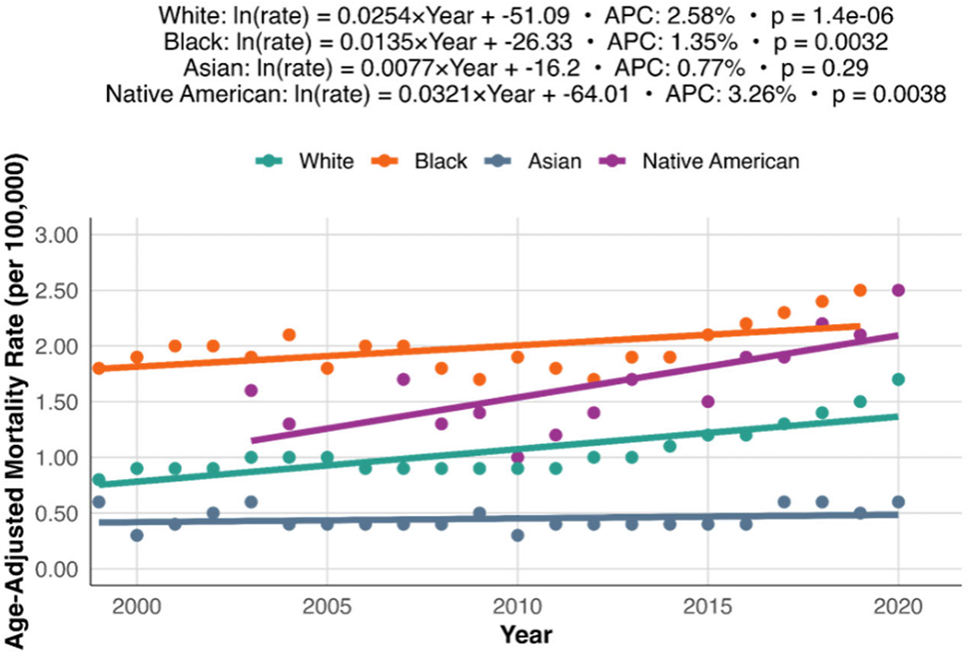
Trends in Osteomyelitis Mortality by Race, United States 1999-2020 Scatter plot and fitted regression lines depicting AAMR trends for osteomyelitis stratified by racial groups. Native American individuals showed the steepest rise in mortality, followed by White and Black individuals, while Asian individuals had stable rates across the study period.

**Figure:**
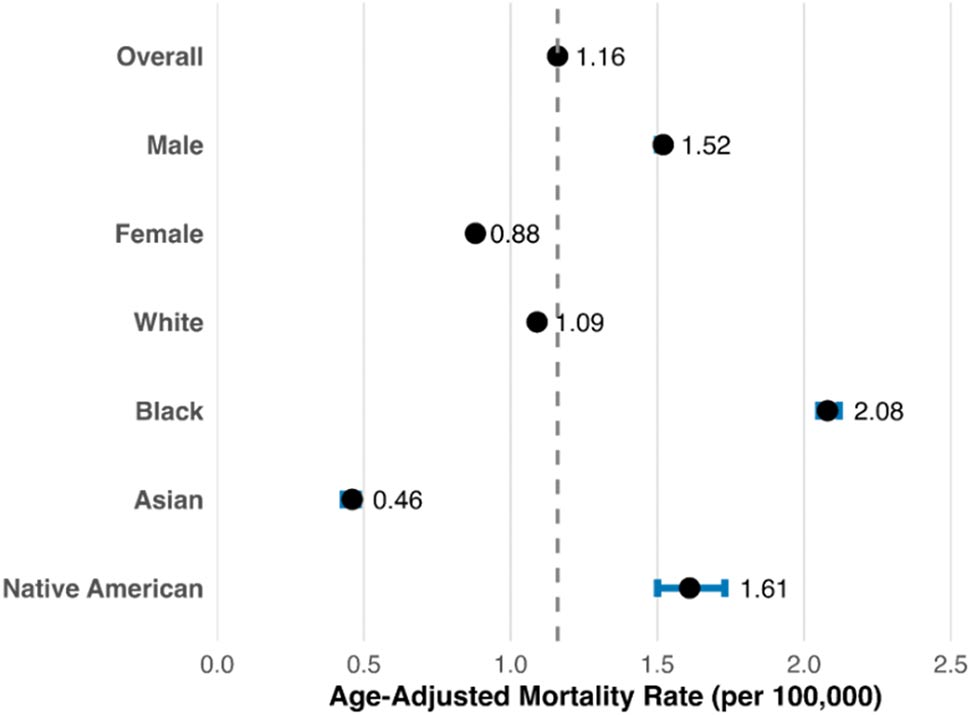
Overall Age-Adjusted Mortality Rates by Demographic Group, United States 1999-2020 Forest plot depicting the overall and demographic-specific age-adjusted mortality rates (AAMRs) for osteomyelitis. Male and Black individuals had the highest AAMRs compared to other groups. Native American individuals also demonstrated elevated AAMRs. Error bars represent 95% confidence intervals.

**Results:**

From 1999-2020, there were 85,474 death certificates with osteomyelitis listed as a contributing factor. The AAMR rose from 0.9 per 100,000 in 1999 to 1.8 per 100,000 in 2020, resulting in an APC of 2.57% (p < 0.01) (Figure 1). AAMR appeared to rise more quickly in male persons, with an APC of 3.1% (p < 0.01) compared to 1.47% in female persons (p < 0.01) (Figure 2). Racial groups also showed differences in their trends in AAMR over the study period (Figure 3). Native American persons showed the greatest increase, with an APC of 3.26% (p < 0.01), followed by White persons with 2.58% (p < 0.01), and Black persons with 1.35% (p < 0.01). Asian persons showed no significant change in their mortality rates over the study period. Overall, male persons demonstrated significantly higher AAMRs than female persons (1.52 per 100,000 vs. 0.88 per 100,000) and Black persons had significantly higher AAMRs (2.08 per 100,000) than Native American (1.61 per 100,000), White (1.09 per 100,000), and Asian persons (0.46 per 100,000) (Figure 4).

**Conclusion:**

Osteomyelitis mortality rates have steadily increased over the past two decades, with marked demographic disparities. These findings underscore the need for improved prevention, earlier diagnosis, and targeted intervention strategies, particularly in high-burden populations.

**Disclosures:**

All Authors: No reported disclosures

